# A real challenge to tackle the overuse of antibiotics in LMIC: A case from Vietnam

**DOI:** 10.1016/j.lanwpc.2022.100650

**Published:** 2022-11-25

**Authors:** Dinh Son Bui, Tuan Nguyen

**Affiliations:** a Centre for Epidemiology and Biostatistics, Melbourne School of Population and Global Health, The University of Melbourne, Melbourne, VIC, Australia; b Social Gerontology Division, National Aging Research Institute, Melbourne, VIC, Australia; c Department of Psychological Sciences, School of Health Sciences, Swinburne University of Technology, Melbourne, VIC, Australia; d Clinical and Health Sciences, University of South Australia, Adelaide, SA, Australia; e Health Strategy and Policy Institute, Ministry of Health of Vietnam, Hanoi, Vietnam

To the editor,

Overuse and/or misuse of antibiotics has long been a public health problem, particularly in many Low-or Middle- Income Countries (LMIC). Antibiotic overuse is a main contributor to increasing rates of antibiotic resistance (AR) which is a real challenge for infectious disease control in coming years.^1^ It also imposes additional costs and increases risk of adverse drug reactions.

A recent paper in the Lancet Regional Health^2^ provides insights into antibiotic overuse in Vietnam as a case study for LMICs. Using robust data from electronic health records for a very large sample (n = 193,010 visits from 112 commune health centres), the authors reported an alarming rate of antibiotic overuse in Vietnamese rural primary care settings. Specifically, 97.0% of individuals with a diagnosis of acute respiratory infections were prescribed with antibiotics. Children were more likely to be prescribed with watch-antibiotics (i.e. a WHO-classified- group of antibiotics prone to development of antibiotic resistance and recommended as the first and second choice for only a limited number of infective syndromes). The authors suggested a high demand for antibiotics from patients, limited capacity of general prescribers (GPs) and unavailability of supporting tests (e.g. to differentiate viral from bacterial infections) as potential determinants of overprescribing. These findings provide an impetus and highlight a need for urgent action to tackle the long term issue of antibiotic overuse and resistance. However, the study only shows the tip of an iceberg and the authors only discussed medical factors. A systemic approach looking at key stakeholders of patients, general prescribers (GPs), medicines dispensers and health and pharmaceutical regulators, and addressing all aspects of the issue including medical, social, cultural, economic, regulatory factors needs to be explored and integrated if effective intervention strategies are to be formulated.

First, the antibiotic overuse comes from patients. The recent Lancet Regional Health study^2^ investigated patient characteristics as determinants of antibiotic choice. However, it failed to discuss determinants of the patient “self-medication”. Antibiotic self-medication in which patients make a diagnosis, “self-prescribe” and purchase antibiotics for themselves without a required prescription from a licensed prescriber, is common in Vietnam.^3^ It is estimated that up to 91% of antibiotics are dispended and consumed through self-medication in this country.^3^ Multiple factors contribute to this issue including limited accessibility to primary care service encompassing unmet capacity of the primary care system, affordability and coverage of the national health insurance, poor regulation enforcement and purchase habits. In addition, retail pharmacies staff also contribute to antibiotic self-medication by directly counselling and supplying antibiotics to patients. Despite regulations on distribution of antibiotics as prescription medicines, antibiotics are still easily accessible from retail pharmacies.^4^ Economic factor may be a main driver of the supply of antibiotics without a prescription at private pharmacies. It is noted that, due to stricter regulations in recent years, there is an increase in the number of retail pharmacies with a pharmacist in charge having a Bpharm (i.e., qualified pharmacist), but many pharmacies, particularly in rural areas, are still managed and operated by individuals with only a certificate or diploma degree in pharmacy (i.e., those with training courses from 1 to 3 years). Even in pharmacies with a qualified pharmacist in charge, it is not uncommon that many are actually operated without the presence of the qualified pharmacist in charge.

Second, the antibiotic overuse is contributed by GP overprescribing. Unfortunately, data on characteristics of prescribers (GPs) were not available in the recent Lancet Regional Health study^2^ to assess the GP determinants of antibiotic overprescribing. The authors recommended training in antimicrobial stewardship and communication skills for GPs and providing GPs in rural care settings with point of care diagnostic tests, specifically C-reactive protein (CRP), to guide antibiotic prescribing. In fact, a trial is undertaking to evaluate impact of CRP point of care (POC) testing in routine care at rural settings in Vietnam.^5^ Even if this trial proves its effectiveness, there are still many hindrances for wide application of CRP testing in clinical practice in Vietnamese rural primary care settings. Such hindrances include additional testing costs, prolonged visits, limited lab technician capacity. These measures only address the medical factors, but the bigger problem is at the system where there is poor enforcement of legislation, lack of transparency and accountability and a high prevalence of corruption that foster prescribing for private gain rather than based on clinical needs.^6^^,^^7^ It is estimated that financial inducements to prescribers may account for 40–60% of the price of off-patent medicines in Vietnam.^7^ In addition, other factors also play a role. A habit of early use of antibiotics for viral infections to prevent secondary bacterial infections is reported. In fact, antibiotics were surprisingly prescribed in 78% of cases with influenza in this study.

In conclusion, in Vietnam, an alarmingly high rate of overprescribing of antibiotics at primary care settings coupling with a high rate of antibiotic self-medication leads to overuse and misuse of antibiotics and their associated consequences including antibiotic resistance. While Vietnam is accelerating its capacity in primary care with a focus on increasing the role and numbers of family doctors, tackling the issue of overprescribing should be seriously considered in parallel. A multimodal intervention strategy addressing issues related to multi-stakeholders including consumers, prescribers, pharmacies, regulators and inspectors, is urgently needed.

## Declaration of interests

None.
